# Culturing Stool Specimens for *Campylobacter* spp*.,* Pennsylvania, USA

**DOI:** 10.3201/eid1803.111266

**Published:** 2012-03

**Authors:** Nkuchia M. M’ikanatha, Lisa A. Dettinger, Amanda Perry, Paul Rogers, Stanley M. Reynolds, Irving Nachamkin

**Affiliations:** Pennsylvania Department of Health, Harrisburg, Pennsylvania, USA (N.M. M’ikanatha, A. Perry);; Perelman School of Medicine, University of Pennsylvania, Philadelphia, Pennsylvania, USA (N.M. M’ikanatha, I. Nachamkin);; Pennsylvania Department of Health, Exton, Pennsylvania, USA (L.A. Dettinger, P. Rogers, S.M. Reynolds)

**Keywords:** stool cultures, gastrointestinal infections, Campylobacter, laboratory practices, bacteria

## Abstract

In 2010, we surveyed 176 clinical laboratories in Pennsylvania regarding stool specimen testing practices for enteropathogens, including *Campylobacter* spp. Most (96.3%) routinely test for *Campylobacter* spp. In 17 (15.7%), a stool antigen test is the sole method for diagnosis. We recommend that laboratory practice guidelines for *Campylobacter* spp. testing be developed.

Clinical microbiology laboratories play a critical role in surveillance for infectious diseases, including recognition of outbreaks and clarification of disease trends over time (*1*). Few studies have examined laboratory testing practices for common enteric pathogens, particularly *Campylobacter* spp*.*, fastidious organisms that can be difficult to detect because of specimen transport and specific culture requirements (*2**–**4*). With the exception of geographic locations included in the Centers for Disease Control and Prevention’s (CDC’s) Foodborne Diseases Active Surveillance Network (*5*), surveillance for *Campylobacter* spp. is largely based on passive reporting without additional confirmation by public health laboratories.

Surveillance for *Campylobacter* spp. in Pennsylvania is limited, and only a fraction of isolates are submitted to the state public health laboratory because there is no regulatory requirement to do so. Because testing practices for enteric infections are not standardized (and largely unknown in Pennsylvania), understanding the methods used to diagnose enteric diseases in clinical laboratories is essential if surveillance programs are to be strengthened. We describe the results obtained from a survey conducted among clinical microbiology laboratories in Pennsylvania to assess laboratory testing practices for enteric pathogens, with an emphasis on *Campylobacter* diagnostics.

## The Study

In November 2010, the Pennsylvania Bureau of Laboratories used an automated laboratory information system to send, by fax, a standardized questionnaire to 176 (86.6%) of the 203 clinical microbiology laboratories in Pennsylvania. The questionnaire assessed selected characteristics of stool-testing practices in calendar year 2009, the type of testing for routine stool specimen workup, use of transport media for stool samples, specimen-processing time, and specific laboratory testing practices for *Campylobacter* spp.

One hundred forty-nine (84.7%) laboratories responded to the survey; 144 were hospital based, 3 were reference laboratories, and 2 were public health laboratories. Hospital laboratories had, on average, 5.7 (range 0–43) full-time equivalent employees, of which 5.4 were certified by a credentialing agency, such as the American Society for Clinical Pathology, to perform clinical microbiology testing. Of the 149 responding laboratories, 107 (71.8%) tested stool specimens for enteric pathogens in house.

In Pennsylvania, all 107 laboratories included *Salmonella* and *Shigella* spp. in the routine testing protocol for enteric pathogens, and 104 (97.2%) routinely included testing for *Campylobacter* spp. (Table 1). Sixty-one (57.0%) laboratories included either *Escherichia coli* O157 cultures or culture plus stool toxin testing. Testing for *Aeromonas* and *Plesiomonas* spp. was included as routine by 57.9% and 55.1% of laboratories, respectively. Most (75.7%) stool specimens were processed within 4 hours after receipt in the laboratory, but only 43.9% of laboratories received specimens in transport media, such as Cary-Blair. Although we did not assess the time from collection of the sample to delivery in the laboratory, given the fastidious nature of *Campylobacter* spp., delays in stool-specimen processing might affect recovery of the organisms, especially if transport medium was not used. Of 107 laboratories in our survey, 99 (92.5%) performed fecal white cell analysis. Fecal white cell analysis has been promoted by some researchers as a useful test for triaging stool samples for culture and for enabling case management decisions (*6*). However, the evidence for using this test in treatment decisions is weak, at best, and it is not recommended for routine use or for decision making regarding type of pathogen or treatment (*7*).

**Table 1:** General laboratory practices for 107 Pennsylvania laboratories performing testing of stool specimens, 2009*

Laboratory practice/method	No. (%) laboratories
Routine stool culture includes the following pathogens	
*Salmonella* spp.	107 (100)
*Shigella* spp.	107 (100)
*Campylobacter* spp.	104 (97.2)
*Aeromonas* spp.	62 (57.9)
*Plesiomonas* spp.	59 (55.1)
*Vibrio* spp.	24 (22.2)
*Yersinia* spp.	38 (35.5)
*Escherichia coli* O157 and STEC stool testing	
Routine *E. coli* O157culture	44 (41.1)
Culture plus Shiga toxin antigen testing	17 (15.8)
Special request *E. coli* O157 culture	47 (43.9)
Special request Shiga-toxin antigen	52 (48.6)
Special request stool culture for the following pathogens	
*Aeromonas* spp.	35 (32.7)
*Plesiomonas* spp.	34 (31.8)
*Vibrio* spp.	75 (70.0)
*Yersinia* spp.	65 (60.7)
Fecal white cell analysis	99 (92.5)
Transport medium†	47 (43.9)
Medium used	
Cary-Blair	41 (87.0)
Not specified	6 (13.0)
No medium used	59 (55.1)
No response	1 (<1.0)
Average time to plating stool specimen after receipt, h	
<4	81 (75.7)
4–8	23 (21.5)
>8	3 (2.8)

*STEC, Shiga toxin–producing *E. coli.* †Laboratories received specimens in transport media >75% of the time.

In a College of American Pathologists Quality Probe (CAP Q-Probe) study conducted in 1996 (*3*), 96% of 601 laboratories that responded to a survey reported including *Campylobacter* spp. as part of the routine stool culture workup. In fact, the data on routine culture workup from the current study look remarkably similar to the data from the CAP Q-Probe survey. The CAP Q-Probe survey also showed that 33.9% of laboratories included cultures for *E. coli* O157. A 1999 CDC survey of stool culture practices by 388 laboratories at 9 FoodNet surveillance sites (*2*) found that most laboratories (97%) included *Campylobacter* spp. in their routine stool culture–testing procedure, but the respondents did not comment on specific laboratory testing protocols. All laboratories in the CDC survey performed cultures for *Salmonella* and *Shigella* spp.; however, only 57% of laboratories routinely tested all stool samples for *E. coli* O157. A CDC survey of 264 clinical laboratories at 5 FoodNet sites during 1996 found several laboratory testing differences in culturing for *Salmonella* spp (*4*).

Among laboratories in the present survey, some variation occurred in the type of culture media used for *Campylobacter* spp. isolation (Table 2), but most laboratories used either cefoperazone-vancomycin-amphotericin agar or *Campylobacte*r blood agar plates (Campy-BAP). Few studies have evaluated multiple media for isolation of *Campylobacter* spp*.*; however, Arzate Barbosa et al. (*8*) showed that Campy-BAP was significantly less sensitive to a charcoal-containing formulation, charcoal-cefoperazone-deoxycholate agar, for isolating *Campylobacter* spp. Two of the laboratories in our survey reported using a charcoal-based medium, Campy charcoal-based selective medium. In a comparison of several media, Endtz et al. (*9*) also found that Campy-BAP was particularly insensitive for detecting *C. coli* isolates.

**Table 2:** *Campylobacter*-specific laboratory practices for 107 Pennsylvania laboratories performing testing of stool specimens, 2009*

Laboratory practice/method	No. (%) laboratories
Included in routine testing	104 (97.2)
Special request culture	1 (0.9)
Culture plus antigen testing	1 (0.9)
Perform culture on positive antigen assay	2 (1.9)
*Campylobacte*r antigen testing only	17 (15.8)
Culture broth enrichment usage	
Yes (9 Campy-Thio, 1 GNB, 4 unspecified)	14 (13.1)
No	89 (83.2)
No response	4 (3.7)
Length of Incubation, h	
24	1 (<1)
48	64 (59.8)
72	33 (30.8)
No response	9 (8.4)
Type of medium used for *Campylobacter* culture	
Campy-BAP	65 (60.7)
CVA	30 (27.8)
Skirrow	2 (1.9)
CSM	2 (1.8)
CCDA or mCCDA	0
Not specified or not cultured	8 (7.5)
Temperature used for culture, °C	
37	3 (2.8)
42	96 (89.7)
Not specified	8 (7.5)
Atmosphere used for culture	
Microaerobic, 5% O_2_	96 (89.7)
10% CO_2_	1 (0.9)
Both	1(0.9)
Not specified	9 (8.4)
Tests used for identification of *Campylobacter* spp.	
Gram stain	96 (89.7)
Oxidase	92 (86)
Catalase	70 (65.4)
Hippurate hydrolysis	51 (47.7)
Naladixic acid/cephalothin disk identification	21 (19.6)
Indoxyl acetate	7 (6.5)
Send to a reference laboratory	6 (5.6)
Other, not specified	33 (30.8)
Performs susceptibility testing	4 (3.7)

*Campy-Thio, *Campylobacter* thioglycollate broth; GNB, gram-negative broth; Campy-BAP, *Campylobacte*r blood agar plates; CVA, cefoperazone-vancomycin-amphotericin; CSM, charcoal-based selective medium; CCDA, charcoal-cefoperazone-deoxycholate agar; mCCDA, modified CCDA.

Several laboratories in our survey used enrichment media for culturing *Campylobacter* spp., although the value of using enrichment media still needs to be addressed (*10*). One laboratory reported using a CO_2_ atmosphere for *Campylobacter* culture rather than microaerobic conditions. Although this usage represents a small proportion of laboratories, suboptimal conditions for isolation of *Campylobacter* spp. will result in false-negative results. Whether this practice is more widespread in laboratories outside Pennsylvania is unknown.

Most laboratories used 42°C for incubating *Campylobacter* cultures, the optimum temperature for the most common campylobacters, mainly *C. jejuni* and *C. coli*. The incubation time before the culture is finalized was 48 hours for 64 laboratories and 72 hours for 33 laboratories. Of note, 1 laboratory incubates the culture for only 24 hours before it reports the results as negative. We also found that laboratory practices vary in performing assays to identify *Campylobacter* once it is isolated. Most laboratories (89.7%) used the Gram stain and oxidase test identify *Campylobacter* spp., but only 51 laboratories (47.7%) used the hippurate hydrolysis test to identify *C. jejuni*. Hippurate hydrolysis is one of the most useful and simplest methods of identifying *C. jejuni* without additional phenotypic testing (*10*). Disk identification methods were used by 21 (19.6%) laboratories, although the usefulness of these tests is limited (*10*). While resistance to antimicrobial drugs is a concern, particularly to fluoroquinolones (*11*), only 4 (3.7%) laboratories tested *Campylobacter* isolates for susceptibility to drugs used for treatment.

In 2009, 18 (16.8%) Pennsylvania laboratories used commercial stool specimen antigen assays for detecting *Campylobacter* spp*.,* and of particular concern, 17 laboratories used these assays in lieu of culture methods. In a previous CDC survey of 388 laboratories concerning practices of stool specimen analysis during 1999, only 1 laboratory used a stool antigen test as a sole diagnostic test for *Campylobacter* spp (*2*). Taken together, these data suggest that antigen testing for *Campylobacter* spp. in stool specimens is increasing as a sole method for diagnosing *Campylobacter* infection.

Although these data represent a cross-sectional survey of the practices at the time of the survey, laboratory procedures for identifying enteric pathogens, such as *Campylobacter*, typically tend to be stable unless the advantages to implementing new methods are apparent. Given the trend observed, we can reasonably conclude that more laboratories may adopt antigen-detection methods other than stool culture as a means of diagnosing *Campylobacter* infection. A 2011 CDC study that evaluated several different *Campylobacter* stool antigen assays concluded, however, that the performance of stool antigen assays was insufficient as a sole diagnostic for *Campylobacter* spp (*12*). An increase in stool antigen testing for *Campylobacter* spp. would affect surveillance data by causing the number of cases to be underestimated because of poor testing sensitivity and may also result in hampering outbreak investigation because of the poor specificity of antigen testing. In some jurisdictions (e.g., Pennsylvania) antigen test results are excluded in criteria for the case definition for *Campylobacter* infections, although other public health jurisdictions include such results. Inconsistencies across states, resulting from conflicting evidence (*12**,**13*), present a challenge in interpreting data on the national level.

## Conclusions

Standardized guidelines for testing enteric pathogens by clinical laboratories and submission of isolates to public health laboratories can enhance surveillance. For example, in 2009, CDC provided testing guidelines for clinical laboratories for *E. coli* O157 and Shiga toxin–producing *E. coli*. These guidelines recommend both antigen and culture testing of samples from patients with acute community-acquired diarrhea (*14*). Since then, the Pennsylvania Bureau of Laboratories has observed an increase of 48% in the number of laboratories that perform toxin antigen testing. In 2011, 32 sites submitted positive toxin broths, compared with 15 sites in 2009. The characterization of these isolates by public health laboratories has improved surveillance data in addition to enhancing outbreak investigations.

In the CDC survey of clinical laboratories, investigators noted that because almost all laboratories routinely test stool samples for *Campylobacter* spp., regional differences in the incidence of culture-confirmed illness were unlikely to be related to laboratory practices (*2*). Of all fecal pathogens, *Campylobacter* spp. are probably the most difficult for clinical laboratories to isolate, and we found some variation in laboratory practices for isolating these pathogens. Using different methods for testing stool specimens for *Campylobacter* spp. would most likely affect surveillance results. Variation in testing methods would also suggest differences in practices for handling and processing specimens, which would, in turn, affect recovery and detection of *Campylobacter* spp. We conclude that variation in practices likely influences surveillance-based data; however, the extent is unknown.

This study suggests that variation in laboratory practices is a potential problem in surveillance for *Campylobacter* spp. in Pennsylvania. Yet, the differences in laboratory practices for *Campylobacter* spp. are unlikely to be unique to Pennsylvania. These factors need to be considered when surveillance data are interpreted and laboratory training programs are devised. Our study also suggests that laboratory practice guidelines for *Campylobacter* testing should be developed.
